# Hydrothermal Surface Engineering of Anodic WO_3_ Photoelectrode by Simultaneous Iron Doping and Fe_3_O_4_/FeWO_4_ Formation

**DOI:** 10.1021/acsami.5c03437

**Published:** 2025-05-08

**Authors:** Piyali Chatterjee, Daniel Piecha, Sebastian Kotarba, Karolina Syrek, Marcin Pisarek, Grzegorz D. Sulka

**Affiliations:** † Department of Physical Chemistry and Electrochemistry, Faculty of Chemistry, Jagiellonian University, Gronostajowa 2, 30-387 Krakow, Poland; ‡ Doctoral School of Exact and Natural Sciences, Jagiellonian University, Lojasiewicza 11, 30-348 Krakow, Poland; § Laboratory of Surface Analysis, Institute of Physical Chemistry, 119463 Polish Academy of Sciences, Kasprzaka 44/52, Warsaw 01-224, Poland

**Keywords:** photoelectrochemical, photoanode, anodic oxidation, tungsten oxide, water splitting, hydrothermal

## Abstract

This study reports a hydrothermal surface modification approach to porous anodized WO_3_ to enhance its photoelectrochemical water oxidation performance. This results in the Fe doping of monoclinic WO_3_ and the simultaneous formation of Fe-containing phases, such as FeWO_4_ and Fe_3_O_4_. The photocurrent generated at the surface-engineered electrodes was double that of pure WO_3_ with long-term stability. The enhancement is attributable to the creation of oxygen vacancies due to Fe doping and the formation of the heterojunction between WO_3_ and FeWO_4_, a p-type semiconductor, which likely improved the charge carrier lifetime and charge transfer properties. Incident photon to current efficiency (IPCE) measurements revealed enhanced visible light performance, supported by the observed red shift in the light absorption edge. This work is one of the few explorations of WO_3_ photoanodes with an opaque metal substrate that involves fabrication of a light-facing overlayer at the surface. Characterization of the fabricated electrodes was carried out using X-ray diffraction (XRD), scanning electron microscopy (FESEM), energy dispersive X-ray spectroscopy (EDS), X-ray photoelectron spectroscopy (XPS), Raman spectroscopy, and diffuse reflectance spectroscopy (UV–Vis DRS). Photoelectrochemical studies were conducted using linear voltammetry, amperometry, and electrochemical impedance spectroscopy (Nyquist, Bode, and Mott–Schottky plots).

## Graphical abstract

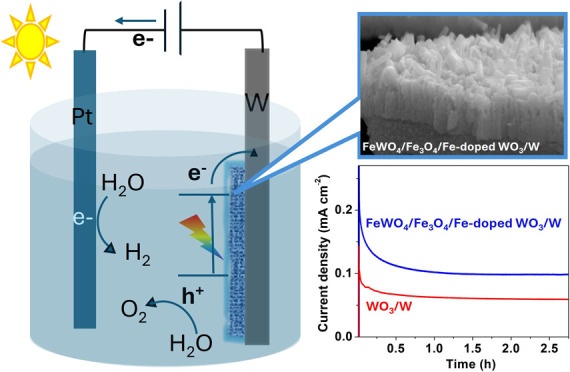

## 1 Introduction

The abundant yet intermittent nature of sunlight necessitates efficient energy conversion and storage mechanisms, with water photoelectrocatalysis for producing portable green hydrogen emerging as a popular solution. A primary challenge in solar water splitting research is the sluggish kinetics of the water oxidation reaction at a photoanode surface, prompting the exploration of numerous efficient materials since the pioneering studies on wide band gap TiO_2_.^1,2^ In photoelectrochemical water splitting, the same material often serves as both the light-absorbing and gas-evolving surface, requiring it to fulfill multiple demanding criteria. Tungsten oxide is a visible light-active, moderate band gap (∼2.7 eV) n-type semiconductor that offers notable advantages, including stability in acidic media, a relatively long (compared to Fe_2_O_3_ for example) hole diffusion length (∼150 nm), and a valence band maximum positioned favorably below the water oxidation potential.^3,4^ Despite these benefits, WO_3_ is susceptible to photocorrosion and has a low absorption coefficient, requiring the use of thicker layers, which increases the risk of unwanted recombination of photogenerated charge carriers. The challenges associated with charge transfer at the substrate/semiconductor interface can be significantly mitigated by directly growing highly crystalline, porous semiconductors on conducting substrates.^5,6^ Doping metal oxides with metal cations improves light absorption, electrical conductivity, and catalytic properties, and is also common in the existing literature on WO_3_ for solar water splitting.^7–9^

To further enhance performance, electrocatalysts grown/deposited on the surface often serve as a suitable co-catalyst for the photoabsorbing primary semiconductor photocatalyst. The electrocatalyst increases the production rate, while the photocatalyst reduces the potential required for electrolysis.^10^ Such surface engineering, particularly when aimed at creating heterojunctions, introduces new interfaces that can potentially hinder the efficient transport of photogenerated charge carriers. However, this challenge can be mitigated through semiconductor doping, which modifies the band gap and/or reduces the material’s ohmic resistance.^7,11^ Alternatively, forming a heterojunction with a material or catalyst that beneficially alters surface chemistry and/or enhances conductivity offers another viable strategy.^12^ Notably, WO_3_/Fe_3_O_4_ heterojunction have been reported to function efficiently as photocatalysts, where Fe_3_O_4_ sensitizes WO_3_ and extends its visible light absorption range.^13,14^ If the conduction and valence band edges of the primary electrode material and the co-catalyst are aligned properly, electron–hole separation is facilitated by the timely transfer of electrons from the anode toward the cathode and holes to water. Incorporating p-type or ferroelectric semiconductors (e.g., CoWO_4_, CaFe_2_O_4_, Fe_3_O_4_, FeWO_4_, etc.) with n-type semiconductors (Fe_2_O_3_, WO_3_, and BiVO_4_) can therefore aid the process by utilizing their built-in potential to drive charge separation.^15–20^ Interestingly, various transition metal tungstates have been previously reported in composites or heterojunctions with WO_3_ due to their similar crystalline structures, particularly the shared chains of WO_6_ groups. Tungstates are chemically robust and offer surface protective roles too.^21–29^ For instance, Zhu et al. demonstrated ∼1.6 times higher photocurrent from a NiWO_4_/WO_3_ heterostructure on FTO glass compared to WO_3_ alone.^21^ Another study also showed performance enhancement using NiWO_4_- and CuWO_4_-modified WO_3_ on FTO glass.^25^ Li et al. reported a photocurrent 5.6 times higher and more stable than that of pristine WO_3_ for hydrothermally grown WO_3_ modified by electrodeposited CuWO_4_/CuO, where p-type CuO acts as a light sensitizer and CuWO_4_ aids in charge separation.^28^ In another study, a 2-fold increase in photocurrent was observed after a multistep Fe-based modification (FeOOH/Fe_2_WO_6_/Fe doping) of hydrothermally grown WO_3_ on FTO glass.^29^ Additionally, FeWO_4_ (E_g_ ∼ 2.0 eV), either pure or containing Fe oxides, has been studied in composites with n-type WO_3_ and WO_*x*_ for applications such as photocatalytic organic pollutant degradation, sensing, and electrocatalytic water splitting.^30–34^ However, despite theoretical predictions suggesting its potential,^35^ the electrocatalytic nature of FeWO_4_ has not yet been utilized as a co-catalyst for WO_3_ photoanodes.^36^

Among various WO_3_ fabrication techniques, anodic oxidation stands out as a highly reproducible and scalable method, where a metal substrate surface is directly converted into the corresponding metal oxide. This process ensures that the oxide layer is strongly adhered to the current collector (metal substrate), resulting in lower interfacial resistances compared to deposition-based electrodes.^37^ This advantage is often underexplored,^38^ especially when compared to widely used transparent conductive glass substrates, where materials are coated or grown on the surface of photoelectrodes.

The opaque nature of the metal substrate in any anodized electrode makes front illumination necessary, and thus only a thin and dispersed overlayer may be made to function in a beneficial way, avoiding shading of the WO_3_ underneath. This is important especially if the overlayer material’s band gap is comparable to WO_3_. Therefore, while extensive studies have focused on pure or doped anodic WO_3_ photoanodes,^37,39,40^ very limited literature exists on co-catalyst-modified anodic WO_3_.^23,38,41^ To address this gap, we aimed to fabricate FeWO_4_ on anodic WO_3_ using hydrothermal method,^30,42^ with the goal of improving efficiency and reducing corrosion of underlying WO_3_. Although hydrothermal synthesis is relatively slow, it offers superior phase control and morphological uniformity compared with other simple wet-chemical methods by tuning parameters such as temperature, time, and solvent. This method allows for the formation of well-crystallized products with moderate scalability, low cost, and operation at moderate temperatures.^43,44^

Generally, doping during anodization requires addition of the dopant precursor in electrolyte or using bimetallic alloys and thus may involve a risk of unwanted defects in the formation of the intended oxide phase. However, our hydrothermal technique can proceed on any pure WO_3_ synthesis route. It is also interesting that our treatment resulted in formation of FeWO_4_–Fe_3_O_4_ phases and simultaneously doped WO_3_ with Fe at the same step.^11^ Notable improvements in photocurrent at low bias and in the visible light range were observed compared to that in pristine WO_3_. These enhancements were correlated with results from X-ray diffraction, X-ray photoelectron spectroscopy, Raman spectroscopy, electrochemical impedance spectroscopy, and changes in the indirect band gap values. To the best of our knowledge, there is no prior report on the use of a hydrothermal technique to engineer the surface of anodic WO_3_ photoelectrodes for better performance.

## 2 Experimental Section

### 2.1 Materials

W metal foil (Goodfellow, 0.2 mm thick, 99.95%), ammonium sulfate ((NH_4_)_2_SO_4_, > 99%, Sigma-Aldrich), ammonium fluoride (NH_4_F, < 99%, Merck), Mohr’s salt/ammonium iron sulfate hexahydrate ((NH_4_)_2_SO_4_·Fe­(SO_4_)·6H_2_O, > 99%, Thermo Scientific), iron­(II) sulfate heptahydrate (FeSO_4_·7H_2_O, > 99%, Sigma-Aldrich), iron­(III) chloride (FeCl_3_, > 99%, Sigma), 25% ammonia (NH_3_, Chempur), hydrofluoric acid (HF, 40%, Merck), iron­(II) chloride tetrahydrate (FeCl_2_·4H_2_O, > 99%, Sigma-Aldrich), sodium hydroxide (NaOH, 98.8%, Chempur), and sodium sulfate (Na_2_SO_4_ > 99%, Sigma-Aldrich) were used without further purification.

#### 2.1.1 Synthesis of WO_3_

WO_3_ was synthesized via anodic oxidation in a two-electrode setup, comprising a W foil (cleaned with ethanol/acetone) as the anode and a Pt wire grid as the cathode (larger than the active area of the anode) positioned 2 cm apart. The W foil was cut into 2 × 1 cm^2^ pieces, and insulating paint was applied, leaving an exposed area of ∼0.5 cm^2^ to define the active area. An aqueous solution of 1 M (NH_4_)_2_SO_4_ and 75 mM NH_4_F was stirred continuously at 20 °C, while a constant voltage of 60 V was applied for 60 min.^45^ After anodization, the electrodes were rinsed three times with deionized (DI) water. Next, the samples were etched in 20% HF at 30 °C under ultrasonication for 15 s, followed by a 5 s rinse in ethanol. The insulating paint was peeled off, and the electrodes were subsequently heated to 500 °C at a rate of 2 °C·min^–1^ in an air muffle furnace and annealed for 2 h. Finally, the samples were allowed to cool naturally to room temperature and stored for future use.^38^ These samples are referred to as WO_3_.

#### 2.1.2 Surface Modification of WO_3_

The annealed WO_3_ electrodes were wrapped with Teflon tape around the edges to maintain a surface metallic for terminals. The samples were clamped and immersed in a beaker containing 30 mL of an aqueous 0.125 mM Mohr’s salt ((NH_4_)_2_SO_4_·Fe­(SO_4_)·6H_2_O) solution (pH = 5.4) under continuous stirring. To adjust the pH, a 25% ammonia (NH_3_) solution was added rapidly, raising the pH to ∼ 8.3. Stirring was maintained for about 45 min, during which the pH came down to ∼8.0. The solution was then transferred to a sealed 50 mL Teflon-lined chamber within a stainless steel autoclave. The electrodes were placed inside in an inclined position, with the WO_3_ surface facing upward to prevent excessive deposition. Hydrothermal treatment was conducted at 180 °C for 6 h in a hot air oven. After slow cooling, the pH of the medium was measured to be ∼ 5.0. The electrodes were rinsed with DI water, air-dried, and annealed at 500 °C for 2 h. These samples, termed WO_3_/NH_3_/Fe, exhibited the best photoelectrochemical performance. A schematic of the procedure is provided in Figure S1 (Supporting Information) along with a possible reaction pathway for FeWO_4_ formation (eqs S1 and S2), provided the thermodynamic conditions are suitable. To give an idea of the optimization of the surface treatment route described above, we provided in Supporting Information some results from WO_3_ samples which were also modified using a similar method but with specific variations, as listed below: (a) replacement of 25% NH_3_ with 1 M NaOH, (b) annealing in vacuum instead of air, (c) use of 25% NH_3_ solution alone without the addition of Fe precursor, (d) substitution of Mohr’s salt with 0.125 mM FeCl_3_, FeCl_2_, or FeSO_4_, and (e) adjustment of the Mohr’s salt concentration from 0.125 to 0.063, 0.25, and 0.5 mM. A summary of all hydrothermal synthesis conditions and their corresponding sample labels is provided in Table S1 (Supporting Information). Preliminary trials indicated that an alkaline pH promotes the formation of smaller particles during synthesis from the Mohr’s salt; therefore, pH 8 was maintained for all samples presented in this work.

#### 2.1.3 Characterization Techniques

Field emission scanning electron microscopy (FESEM) images were obtained by using a Hitachi S-4700 microscope equipped with an X-ray detector for energy dispersive spectroscopy (EDS, Thermo Noran System 7), operated at an acceleration voltage of 20 kV. The samples were sputter-coated with Au using a sputter current of 10 mA for 120 s, without any precleaning step. The corresponding FESEM images were analyzed by using ImageJ software. The UV–visible diffuse reflectance spectroscopy (UV–Vis DRS) was performed in the 200–800 nm wavelength range using a PerkinElmer Lambda 750S spectrophotometer. X-ray powder diffraction (XRD) measurements were carried out on a Malvern Panalytical Aeris diffractometer in the 2θ range of 10°-80°, with a step size of 0.02° and a scan rate of 2.8° min^–1^. Cu Kα radiation (1.54 Å) was employed during acquisition with operating conditions set at 40 kV and 15 mA. X-ray photoelectron spectroscopy (XPS) was conducted on a Microlab350 spectrometer with a 20 W X-ray gun and an Al X-ray source (1486.6 eV). XPS spectra were analyzed using Thermo Avantage software (version 5.9911, Thermo Fisher Scientific), with the C 1s adventitious carbon peak at 284.6 eV used as a reference. A Horiba Xplora Plus Raman microscope with an incident laser of 532 nm wavelength operating at low intensity (1% of nominal 100 mW power) was used for Raman measurements. The laser beam was focused on the sample using a 100× objective (Olympus MPlan N, N_A_ = 0.9). Raman wavenumber shifts in the range of 0–1200 cm^–1^ were recorded. All photoelectrochemical studies, except impedance vs potential measurements (Mott–Schottky plots), were performed using a PalmSens4 potentiostat and a Teflon-based three-electrode cell with a quartz glass window. Mott–Schottky measurements were carried out using a Biologic SP-300 or VMP-300 potentiostat. The counter and reference electrodes were a platinum foil and saturated calomel electrode (SCE), respectively. The separation distances between the three electrodes were kept nearly constant.

A 0.1 M Na_2_SO_4_ solution was used as the electrolyte in all experiments, as both FeWO_4_ and WO_3_ are fairly stable at pH < 7. Linear sweep voltammetry (LSV) curves were recorded at a slow scan rate (5 mV s^–1^) to minimize the capacitive dark current. The measurements were conducted under chopped illumination with 5 s on–off cycles. The potential window ranged from just below the onset potential to beyond the water oxidation potential at 1.23 V vs RHE (∼0.6 V vs SCE). The Nernst equation used for the conversion from the SCE to RHE scale is given in eq S3 of Supporting Information. Nyquist plots were derived from electrochemical impedance spectroscopy (EIS) measurements conducted over a frequency range of 0.1 Hz - 100 kHz with a 10 mV AC amplitude. Solar simulated illumination at 100 mW cm^–2^ power was provided by a 150 W Xe arc lamp (Instytut Fotonowy, Poland). Charge separation and charge injection efficiencies were measured by adding 0.1 M Na_2_SO_3_ to 0.1 M Na_2_SO_4_. The incident photon to current efficiency (IPCE) measurements were performed using a specialized photoelectric spectrometer equipped with a monochromator (Instytut Fotonowy, Poland). The IPCE plots were derived from photocurrent data recorded at 1.2 V vs RHE, using monochromatic light with a stepwise increase of 10 nm in the wavelength range of 300–480 nm.

## 3 Results and Discussion

The initial stage of the research involved the selection of the best hydrothermal modification route for anodic WO_3_ which has been discussed to some extent in the Supporting Information. Figures S2 and S3 (Supporting Information) present the top-view morphology of anodic WO_3_ samples modified under various hydrothermal reaction parameters and an annealing atmosphere. The fabricated photoanodes were tested in the three-electrode configuration to compare their photoelectrochemical performances (see Figure S4, Supporting Information) and thus optimize the hydrothermal surface engineering protocol.

The top surface of pristine porous anodic WO_3_ is shown in Figure 1a, which represents a typical morphology formed by the anodization method employed.^45^ The cross-sectional view of this electrode, provided in the inset, distinctly shows the anodic layer (marked green) and the thermally oxidized layer (marked blue) of WO_3_, with measured thicknesses of about 300 and 700 nm, respectively, across different samples. Figure 1b displays a top view of the WO_3_/NH_3_/Fe sample, revealing an irregular plate-like morphology. This morphology is better understood in the lateral view in the inset showing plates of width <70 nm. A comparison of the two cross-section images highlights the significant transformation of the anodic WO_3_ layer into WO_3_/NH_3_/Fe. Despite this transformation, the total height (marked yellow) of the structure above the tungsten substrate remains comparable to that of pristine WO_3_.

**Figure 1:**
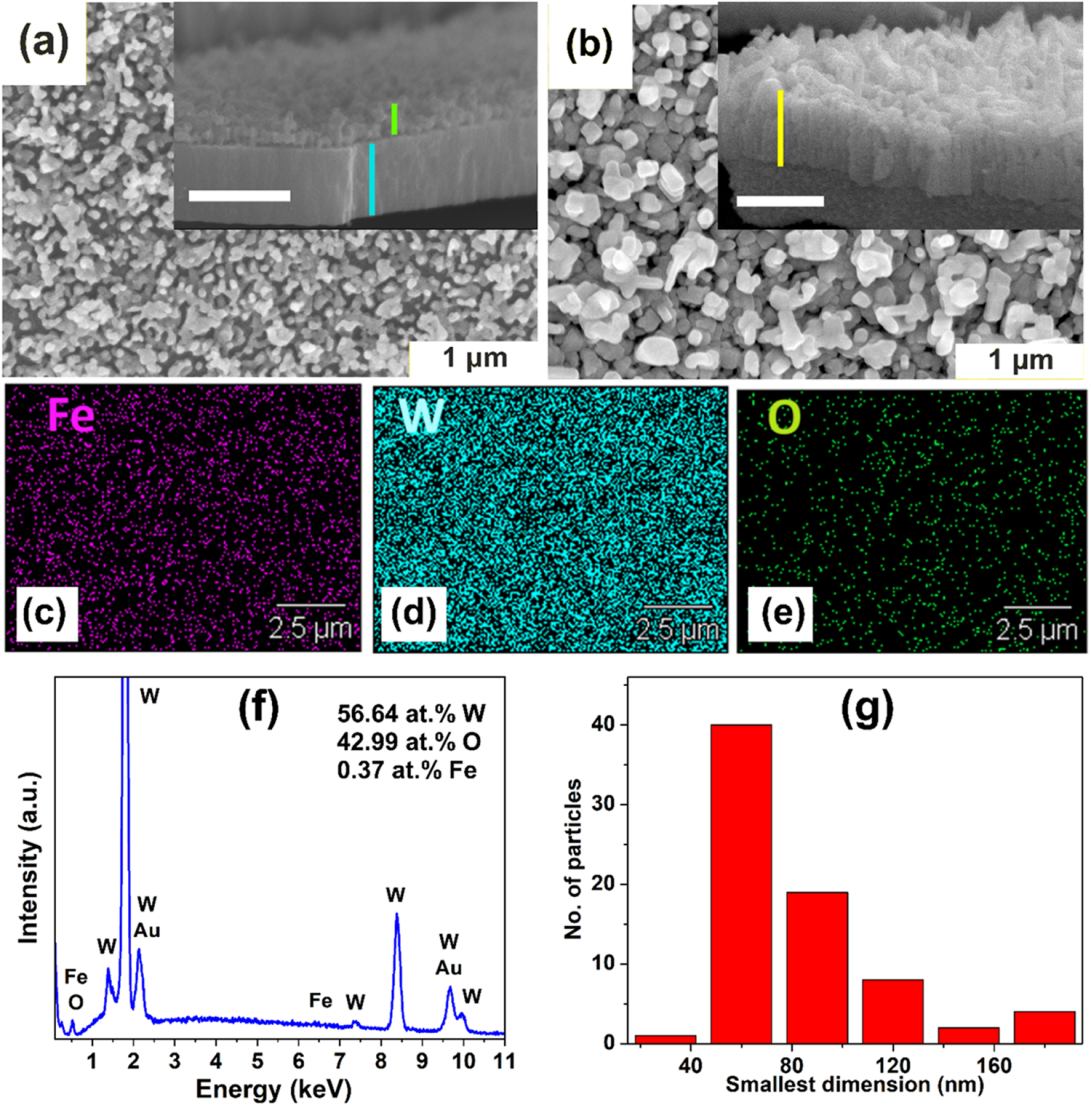
FESEM images of (a) WO_3_ (top view) and (inset) its cross-sectional view with 1 μm scale bar. (b) WO_3_/NH_3_/Fe (top view) and (inset) its cross-sectional view with 1 μm scale bar. (c–e) Representative elemental mapping for Fe, W, and O on the WO_3_/NH_3_/Fe electrode surface. (f) Corresponding areal EDS spectrum for a WO_3_/NH_3_/Fe sample. (g) Particle size histogram of WO_3_/NH_3_/Fe.

Figure 1c–e shows the elemental mapping of WO_3_/NH_3_/Fe obtained by EDS, revealing a mostly uniform coverage of the WO_3_ surface with Fe. As expected, the EDS spectra in Figure 1f show the presence of W and O, along with Au (from sputter coating) and trace amounts of Fe. The absence of any noticeable N or S signal from the precursors used indicates a clean synthesis procedure. The stoichiometric 1:3 ratio (or at least a higher at.% of O compared to W) in WO_3_ is not observed, which is understandable due to the presence of metallic W beneath the anodic oxide layers. The average Fe:W atomic ratio is ∼1% in all samples, indicating very dispersed incorporation of Fe on or within WO_3,_ as desirable. Figure 1g shows the particle size histogram of WO_3_/NH_3_/Fe. It indicates that the median length of the smallest dimension observed in the top view of the particles on the WO_3_/NH_3_/Fe surface is 56 nm, while the mean length is 69 nm.

The X-ray diffractograms for the optimized samples annealed in air (WO_3_/NH_3_/Fe) and in vacuum (WO_3_/NH_3_/Fe (vac)) are plotted in Figure 2a along with that of pristine WO_3_ and standard reference databases for comparison. The sharp peaks observed at 2θ = 40.5, 58.5, and 73.4° are attributed to the cubic W foil substrate, corresponding to the (110), (200), and (211) planes, as matched with PDF card no. 00-001-1203. For WO_3_, the major peaks observed are characteristic of the stoichiometric monoclinic WO_3_ phase (PDF card no. 04-019-8947). For WO_3_/NH_3_/Fe, all of the major peaks corresponding to another monoclinic phase (PDF card no. 00-030-1387) can also be identified. This phase has the empirical formula WO_2.92_ and is one of the tungsten oxide polymorphs.^46^ For doping WO_3_ by Fe^2+^ precursor, a slight shift of the most intense stoichiometric WO_3_ peak to higher angle has been found in literature as has also happened in our case (∼0.4° shift).^47^ Since the W:O stoichiometry is not identical across these anodic oxide materials, the observed shift can only be partially attributed to the slightly smaller covalent radius of Fe compared to W. Fe doping likely causes a contraction of the WO_3_ lattice parameters, which, in turn, leads to a shift in peak positions according to Bragg’s law of diffraction, along with changes in peak intensity ratios. For example, the ratio of the peak heights at ∼23 and 24° decreases from 9.3 in pristine WO_3_ to 6.2 in the WO_3_/NH_3_/Fe sample.^48,49^ However, very prominent shifts of the maxima of the most intense peak of WO_3_ cannot be expected even for Fe doping in WO_3_/NH_3_/Fe, due to the similar ionic radii of W^6+^ (0.62 nm) and the Fe ions (Fe^4+^ – 0.39 nm, Fe^3+^ – 0.64 nm, Fe^2+^ – 0.77 nm).^50^ In the expanded plot in Figure 2b, minor peaks at 2θ = 30.7, 19.0, and 36.5° correspond to the (111), (100), and (021) planes of monoclinic FeWO_4_ (PDF card no. 01-074-1130), while the peak at 35.6° may originate from the most intense reflection of Fe_3_O_4_ (PDF card no. 01-080-6406). The peaks corresponding to FeWO_4_ are more intense and easily identifiable for the vacuum-annealed WO_3_/NH_3_/Fe (vac) sample and FeWO_4_ appears to have undergone partial oxidative decomposition when annealed in air. The average crystallite size of FeWO_4_ nanoparticles in the overlayer is estimated to be 18 nm, as calculated using the Debye–Scherrer equation (eq S4, Supporting Information).^49^

**Figure 2:**
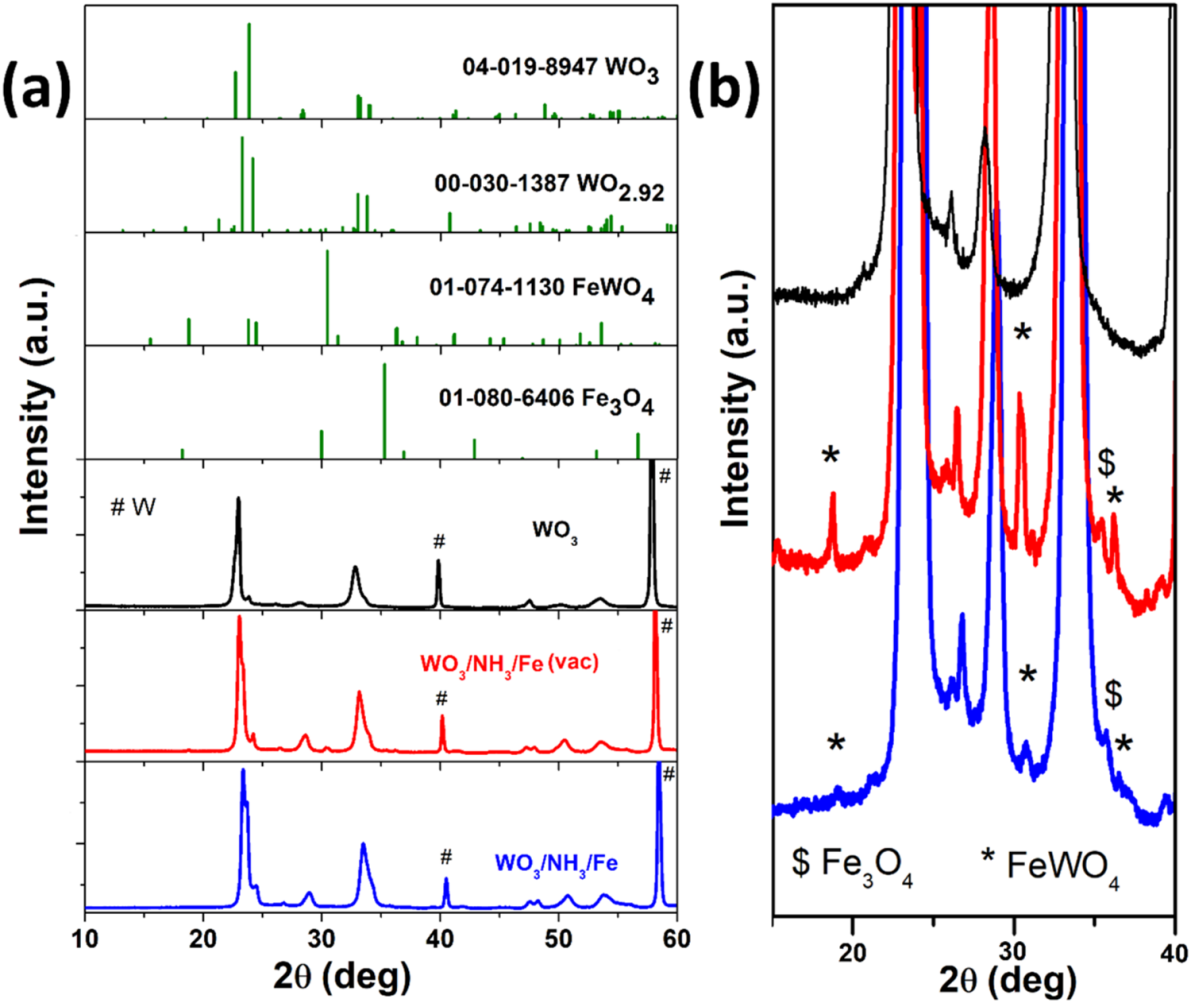
(a) X-ray diffractograms of pristine WO_3_ along with hydrothermally modified WO_3_/NH_3_/Fe and WO_3_/NH_3_/Fe (vac) samples, in comparison to standard PDF5+ database patterns. (b) Stacked and intensity expanded diffractograms of WO_3_, WO_3_/NH_3_/Fe (vac), and WO_3_/NH_3_/Fe in the 15–40° range.

X-ray photoelectron spectroscopy was used to investigate the surface changes in WO_3_ after hydrothermal treatment. The high-resolution W 4f spectra in Figure 3c show a distinct peak shift of ∼0.3 eV in the binding energy for 4f_7/2_ and 4f_5/2_ toward lower values in WO_3_/NH_3_/Fe compared to WO_3_ (Figure 3a). This shift was observed for doping by atoms of lower valency, such as Fe^2+^/Fe^3+^ substituting for W^6+^.^51–53^ The contribution from W^5+^ or other WO_*x*_ species, present only in WO_3_/NH_3_/Fe, is relatively weak compared to that of W^6+^ but suggests the presence of oxygen vacancies and supports the WO_2.92_ phase detected by XRD. The deconvoluted O 1s spectra in Figure 3b show the contribution of lattice oxygen (∼530 eV) and adsorbed oxygen (∼532 eV) in WO_3_. However, a significant contribution from oxygen vacancies or oxygen-deficient regions (∼531 eV) is observed only in WO_3_/NH_3_/Fe, as shown in Figure 3d. The low binding energy component is directly related to the stoichiometry of the compound, while the high binding energy component arises from loosely bound oxygen, which cannot be entirely removed by annealing. Furthermore, the O 1s peak in WO_3_/NH_3_/Fe is shifted by ∼0.5 eV toward lower binding energies, suggesting possible Fe doping.^54,55^ The deconvoluted Fe 2p spectra in Figure 3e confirm the presence of both Fe^3+^ and Fe^2+^ oxidation states along with their corresponding satellite peaks. The Fe 2p_3/2_ and 2p_1/2_ peaks are separated by ∼14 eV, with an area ratio of about 2:1, as expected. The Fe^3+^ species may possibly originate from Fe_2_WO_6_, Fe_3_O_4_, or Fe_2_O_3_, while Fe^2+^ could be attributed to FeWO_4_ and Fe_3_O_4_.^30^ However, the XRD patterns specifically indicate the formation of FeWO_4_ and Fe_3_O_4_.

**Figure 3:**
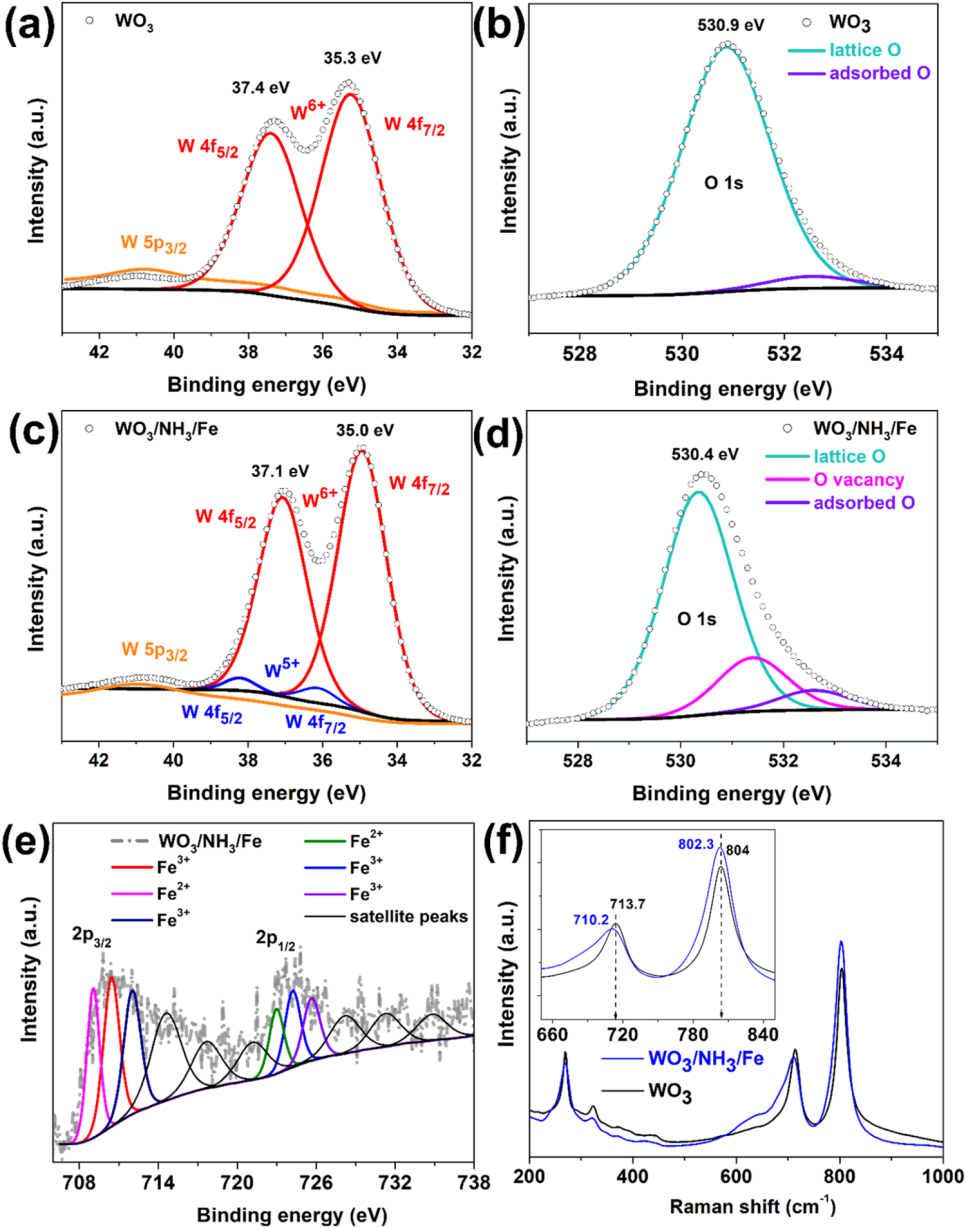
(a, b) High-resolution XPS spectra of WO_3_ for (a) W 4f and (b) O 1s. (c–e) High-resolution XPS spectra of WO_3_/NH_3_/Fe for (c) W 4f, (d) O 1s, and (e) Fe 2p. (f) Raman spectra of WO_3_ and WO_3_/NH_3_/Fe.

The Raman spectra of WO_3_/NH_3_/Fe and WO_3_, shown in Figure 3f, reveal bands at 270, 323, 713, and 803 cm^–1^ that are characteristic of monoclinic WO_3_ vibration modes. The first two bands correspond to O–W–O bending modes, while the next two are associated with O–W–O stretching modes.^56^ A broad hump around 650 cm^–1^ may be attributed to the Fe_3_O_4_ phase in WO_3_/NH_3_/Fe, though it is not prominent due to the low amount of Fe, as suggested by the XRD data. The band at 713 cm^–1^ shifts by 3 cm^–1^ toward lower wavenumbers in WO_3_/NH_3_/Fe, as expected due to low Fe doping.^57^ The band at 804 cm^–1^ shifts slightly further to 802.3 cm^–1^, which is consistent with existing literature on Fe-doped WO_3_.^50^

LSV curves (Figure 4a) were recorded for the optimized air-annealed WO_3_/NH_3_/Fe sample, and presented alongside that from WO_3_.^41^ The WO_3_/NH_3_/Fe sample demonstrated a photocurrent of up to 0.43 mA cm^–2^ at 1.23 V vs RHE, compared to 0.34 mA cm^–2^ for pristine WO_3_. This is undoubtedly a result of doping of the semiconductor layer and the formation of new FeWO_4_ and Fe_3_O_4_ phases on the surface of the anodic WO_3_. The WO_3_/NH_3_/Fe sample exhibited nearly double the photocurrent at lower biases (up to 1.0 V vs RHE) compared to WO_3_, although WO_3_ remained competitive at higher potentials. The performance comparison of these two with other samples prepared with slight variations in the synthesis route is provided in Figure S4 (Supporting Information) but is discussed here to understand better the causes behind the superiority of the best-performing material. In all of the data, the dark current was negligible across the entire potential range, indicating the absence of corrosion or significant electrocatalysis in 0.1 M Na_2_SO_4_. First, the effects of varying the molarity of the overlayer precursor (0.063, 0.125, 0.25 mM Mohr’s salt) are compared in Figure S4a (Supporting Information). The samples synthesized with very low molarity of 0.063 mM performed worse than pristine WO_3_ at higher potentials possibly because the pH set by 25% NH_3_ was insufficiently utilized by the Mohr’s salt, increasing the likelihood of WO_3_ surface corrosion. Also, a higher molarity of 0.25 mM resulted in low photocurrents in the entire potential window. Beyond its intended role in co-catalysis and light sensitization, a thicker low-band gap overlayer might have shaded the underlying WO_3_ from UV light or introduced unfavorable deep defect levels, promoting charge trapping and recombination. Figure S4b (Supporting Information) demonstrates the effect of different Fe precursors, Fe­(III) chloride and Fe­(II) sulfate, on photocurrent, compared to the superior Mohr’s salt at an equal concentration of 0.125 mM. This could be a direct result of the smaller particle sizes of the overlayer in the case of Mohr’s salt. Figure S4c (Supporting Information) compares electrodes annealed in vacuum and air for equal duration and at the same temperature. Vacuum-annealed samples perform worse than their air-annealed counterparts but still showed better PEC activity than pure WO_3_. These studies support the idea that an optimized thin co-catalyst layer is necessary to translate surface properties into higher photocurrent. On the other hand, an excessive amount of Fe compounds on the WO_3_ surface ultimately have a negative impact on photocurrent in a system as this which requires front illumination. Additionally, a control electrode, where NH_3_ was used to set the pH at 8 during hydrothermal treatment without adding Fe precursors, performed worse than pristine WO_3_. This suggests that the observed photocurrent enhancement is not due to N doping by NH_3_ (as confirmed by EDS, Figure 1f) and that alkaline media degrade WO_3_ in the absence of other reactants. It is also observed that replacing NH_3_ with NaOH to achieve the pH = 8 reaction medium (for 0.125 mM Mohr’s salt) led to a severe deterioration in photocurrent, likely due to differences in reaction kinetics. Notably, the onset potentials remained similar, ruling out the contribution from improved visible light absorption as the primary cause of the higher photocurrent.

**Figure 4:**
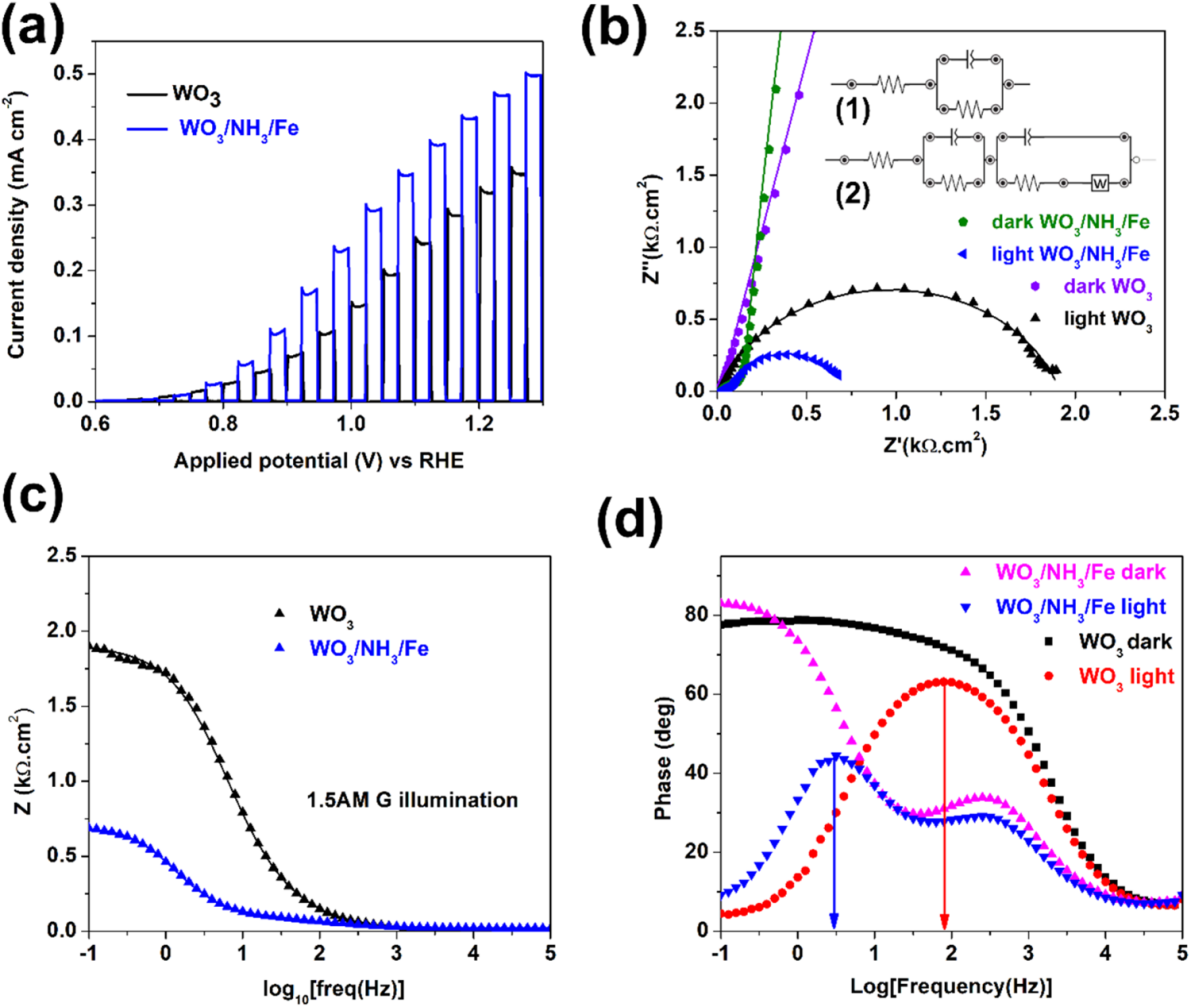
(a) Chopped LSVs under 1.5 AM G illumination for WO_3_ and WO_3_/NH_3_/Fe. (b) Nyquist plots for the electrodes in the dark and under illumination (1.5 AM G) at 1.0 V vs RHE bias. Symbols depict experimental data and solid line curves follow the fitted data. Inset shows the equivalent circuit model for (1) WO_3_ and (2) WO_3_/NH_3_/Fe. (c) Bode impedance plot under illumination and (d) Bode phase plots recorded in the dark and under illumination for WO_3_ and WO_3_/NH_3_/Fe at 1.0 V vs RHE.

To further investigate the electron transfer mechanism, electrochemical impedance spectroscopy was performed. To eliminate the effect of minor differences in the electrode area, the data were normalized. The fitted Nyquist plots, under both dark and illuminated conditions, are presented in Figure 4b for WO_3_ and WO_3_/NH_3_/Fe at a potential (1.0 V vs RHE) significantly above the onset. At this potential, charge transfer occurs without the pronounced effects of radiative recombination, allowing for more reliable equivalent circuit fitting. The corresponding fitted Bode plots under illumination has also been provided in Figure 4c to highlight the goodness of fitting over the full frequency range investigated. Given as the inset of Figure 4b, the circuit (1) for porous WO_3_ features a single R-C loop, whereas that of WO_3_/NH_3_/Fe (2) exhibits an additional loop corresponding to the surface layer.^58^ A Warburg impedance component is also included to account for diffusion effects and is supported by the low-frequency phase plot features observed for WO_3_/NH_3_/Fe (Figure 4(d)). Under illumination, the charge trapping resistances for WO_3_/NH_3_/Fe and WO_3_ are lower than those under dark conditions. Smaller charge transfer resistance at the semiconductor/electrolyte interface, as estimated from the semicircular arc diameter, was observed for WO_3_/NH_3_/Fe. The equivalent circuit under illumination and relevant fitting parameters are summarized in Table S2 (Supporting Information). Notably, the interfacial charge transfer resistance (R_CTinterface_) of pristine WO_3_ under illumination decreased by 3.4 times with surface modification (i.e., for WO_3_/NH_3_/Fe) indicating improved charge separation and suppression of radiative electron–hole pair recombination. This happens due to the change of the chemical nature of the WO_3_ surface by co-catalysts or by improving the conductivity and reducing ohmic resistance of the photoelectrode. The results suggest that the oxygen vacancy-rich WO_3_/NH_3_/Fe surface has beneficial surface defects that can act as active catalytic sites for water oxidation.

The Bode phase plots recorded in the dark and under illumination for WO_3_ and WO_3_/NH_3_/Fe samples as presented in Figure 4d show that the frequency (f) of the prominent peak in the low-frequency range under illumination is lower for WO_3_/NH_3_/Fe compared to WO_3_. This low-frequency range corresponds to charge transfer at the semiconductor/electrolyte interface. The reduced frequency indicates a longer charge carrier lifetime (τ = 1/2πf) for WO_3_/NH_3_/Fe. A longer charge carrier lifetime suppresses recombination, enhances separation efficiency, and consequently can contribute to a higher photocurrent. This improvement can likely be attributed to built-in internal electric field at the p-type FeWO_4_/n-type Fe doped WO_3_ heterojunction,^17^ considering that the amount of the Fe_3_O_4_ phase detected is much less than FeWO_4_.

The amperometric photoresponse of WO_3_/NH_3_/Fe under 3 h of simulated solar illumination at 1.0 V vs RHE is shown in Figure 5a. The initial transient photoresponse spike quickly decays due to the competing effect of recombination and charge transfer but eventually stabilizes into a steady state for both WO_3_/NH_3_/Fe and WO_3_. The steady-state photocurrent value at the end of the illumination period is 1.7 times higher for WO_3_/NH_3_/Fe compared to WO_3_, indicating greater potential for efficient long-term use. Various processes may occur during operation, such as the formation and degradation of peroxo- species, Na^+^ intercalation/deintercalation, healing or expansion of surface defects (e.g., oxygen vacancies), oxide dissolution, and oxidation of the underlying W metal.^59–61^ The prolonged performance tests indicate that an equilibrium is eventually established between detrimental photocorrosion and beneficial water oxidation activity. However, while hydrothermal treatment helps to maintain the stability of WO_3_, it does not lead to a significant enhancement. Figure S4d (Supporting Information) presents current vs time curves recorded at a higher potential of 1.2 V vs RHE over a few minutes of illumination for WO_3_/NH_3_/Fe, WO_3_/NH_3_/Fe (vac), WO_3_/NaOH/Fe, WO_3_/NH_3_, and WO_3_. Among these, even if optimized WO_3_/NH_3_/Fe has the highest magnitude of photocurrent, stability is achieved quicker for WO_3_/NH_3_/Fe (vac) possibly due to the protective role on WO_3_ from having more FeWO_4_ (as indicated by XRD peak intensity in Figure 2b). Figure S5 in Supporting Information presents the data for 30 min of illumination at 1.0 V vs RHE on a different batch of these two samples showing almost double photocurrent when stabilized. Figure 5b demonstrates the applied bias photon to current (ABPE) efficiency of WO_3_/NH_3_/Fe in comparison to WO_3_, assuming a negligible corrosion current and unity faradaic efficiency. The ABPE% was calculated using eq S5 detailed in Supporting Information. The efficiency nearly doubles at ∼0.9 V vs RHE, highlighting the promising utility of the modified photoanodes at low bias. Furthermore, we have studied the charge injection (surface charge separation) and bulk charge separation efficiencies following an established protocol reported in the literature, and the obtained results are presented in Figure S6a,b respectively.^62^ Surface charge recombination is significantly suppressed in the presence of a hole scavenger such as Na_2_SO_3_, while bulk charge separation remains largely unaffected. Therefore, charge injection efficiency was calculated as the ratio of the photocurrent density for water oxidation in 0.1 M Na_2_SO_4_ to that obtained for sulfite oxidation in 0.1 M Na_2_SO_4_ containing 0.1 M Na_2_SO_3_. A higher photocurrent in the presence of sulfite compared with that in its absence indicates greater recombination loss before the photogenerated holes can participate in water oxidation. The WO_3_/NH_3_/Fe electrode exhibits moderately improved charge injection efficiency compared to pristine WO_3_, suggesting enhanced hole transfer from the electrode surface to the electrolyte. The calculation method for charge separation efficiency is provided as eq S6 in Supporting Information.^62^ Notably, the efficiency of WO_3_/NH_3_/Fe is higher and increases with applied potential, supporting conclusions drawn from impedance spectroscopy that the heterojunctions formed contribute to an internal electric field that facilitates faster electron–hole separation, thereby minimizing recombination and improving light energy utilization.

**Figure 5:**
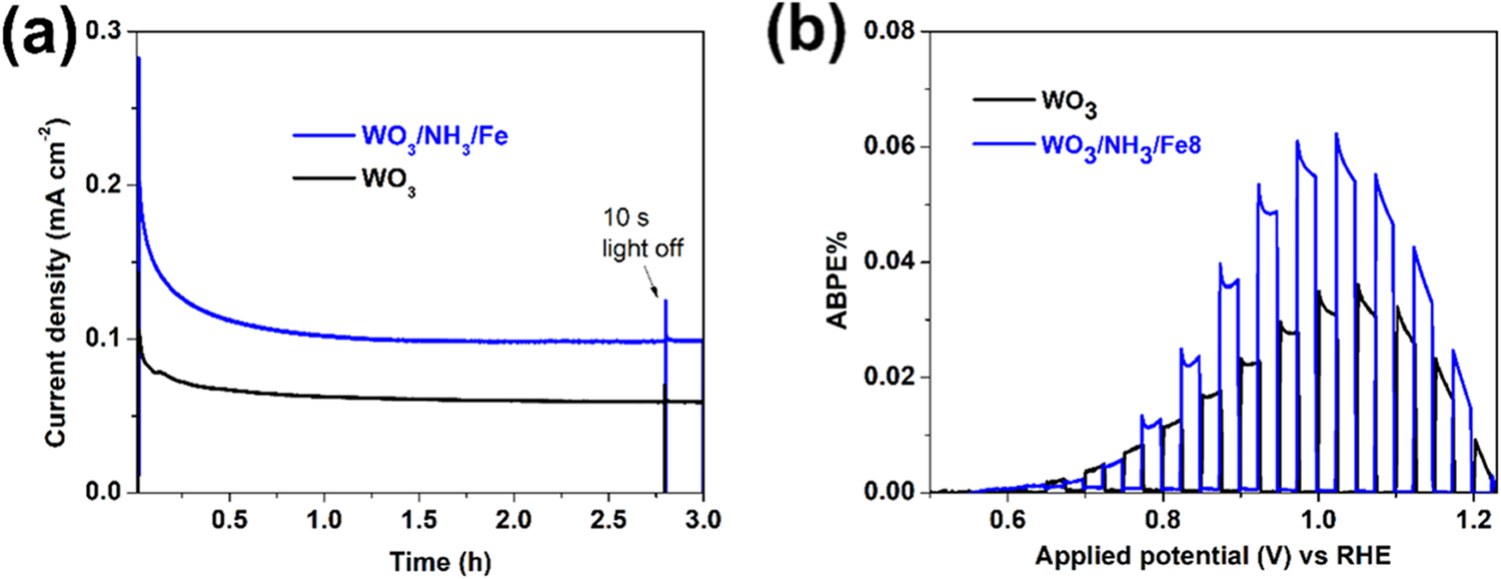
(a) Current vs time curves recorded at 1.0 V vs RHE, and (b) ABPE plots for WO_3_ and WO_3_/NH_3_/Fe materials.

Figure 6a presents the Tauc plots derived from UV–Vis DRS, following the Kubelka–Munk transformation of the reflectance data.^40^ The Tauc equation S7 used to plot and estimate the indirect band gaps has been described in the Supporting Information. The indirect band gaps of WO_3_, and WO_3_/NH_3_/Fe were found to be ∼2.96 and 2.77 eV, respectively. This indicates only a slight shift in the absorption edge, possibly due to VB (valence band maxima) coming closer to CB (conduction band minima) on Fe doping and the sensitization by low band gap phases (Fe_3_O_4_, FeWO_4_), contributing minimally to visible light absorption. Figure 6b,c demonstrates the IPCE plots in different wavelength ranges. The IPCE % has been calculated from photocurrent data using eq S8 described in Supporting Information. It is evident in Figure 6b that both WO_3_ and WO_3_/NH_3_/Fe samples exhibit the highest conversion efficiency under ultraviolet light (∼300 nm), with WO_3_/NH_3_/Fe significantly outperforming WO_3_. Additionally, the IPCE% data in Figure 6c for 400–480 nm wavelength highlight a much higher performance for WO_3_/NH_3_/Fe compared to WO_3_ under visible light. It is not only due to the lower optical band gap but also probably due to the reduced recombination of charge carriers probably. This is much better than existing report of electrodeposited Fe_2_O_3_ on anodic WO_3_ and thus may suggest superiority of Fe doping and interfacing with FeWO_4_ and Fe_3_O_4_ for anodic WO_3_ sensitization over use of only Fe oxide cocatalysts.^38^ This suggests that WO_3_/NH_3_/Fe has a better ability to utilize the visible range of the solar spectrum, pointing toward its applicability under visible light sources such as light emitting diodes (LEDs).

**Figure 6:**
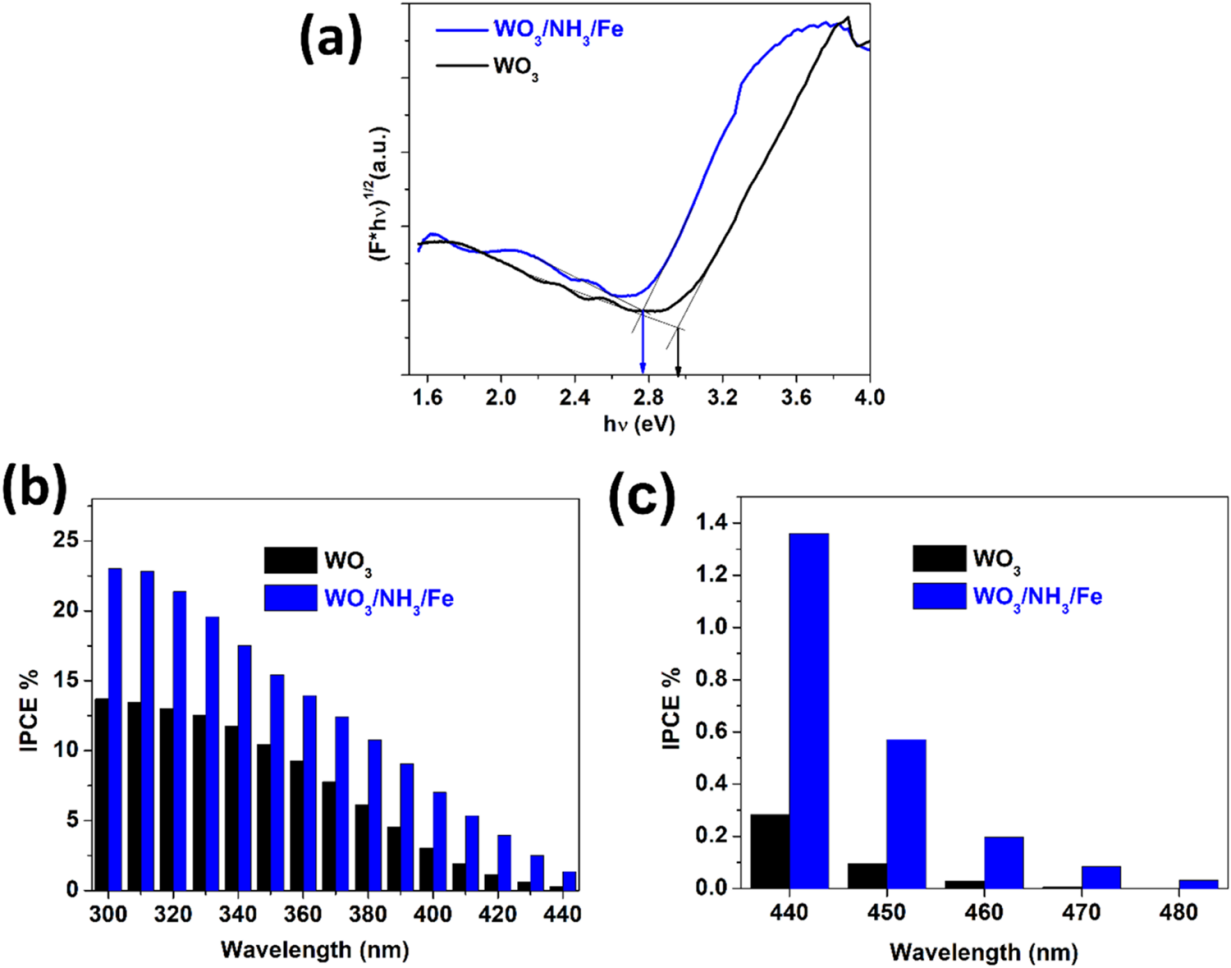
(a) Tauc plots derived from the Kubelka–Munk transformation of UV–Vis DRS data to determine the indirect band gap of WO_3_ and WO_3_/NH_3_/Fe. (b-c) IPCE plots for WO_3_ and WO_3_/NH_3_/Fe at 1.2 V vs RHE for the (b) 300–440 and (c) 440–480 nm wavelength range.

Additionally, impedance vs potential data were recorded in the dark, and the corresponding Mott–Schottky plots (C^-2^ vs potential) at fixed frequencies of 500 and 1000 Hz are shown in Figure 7a. The governing Mott–Schottky eq S9 is provided in the Supporting Information.^22^ The flat band potential V*_fb_* is around 0.4 V vs RHE across different frequencies for both the samples and is just below the onset potential (∼0.5 V vs RHE) observed in the LSV curves as expected. The positive slopes of the linear portion of the Mott–Schottky plots for both WO_3_ and WO_3_/NH_3_/Fe confirm the predominantly n-type semiconductor nature of the primary WO_3_ material. The slope (m) of the linear portion of the plot is indirectly proportional to the value of donor density N*_D_*.^40^ The N_D_ for WO_3_ is therefore significantly lower than that for WO_3_/NH_3_/Fe, as evident from the steep slope and correlated to literature report of Fe-doped WO_3_.^52^ This increased donor concentration which may contribute to enhanced catalytic activity could arise from the observed oxygen vacancies^54^ on aliovalent doping and built-in electric field at the heterojunction.^63^ Surface oxygen vacancies created in WO_3_ can facilitate water molecule adsorption, potentially enhancing the water oxidation kinetics. However, these vacancies can also localize charge carriers, reducing electronic conductivity and promoting recombination of photogenerated holes before they contribute to the desired reaction. Introducing transition metal dopants like Fe can mitigate these issues. The nonlocalized 3d orbitals of Fe may passivate low-energy defect traps, improving charge transport. Additionally, the Fe dopant and FeWO_4_ co-catalyst can passivate intrinsic surface/bulk defects commonly present in anodic tungsten oxide (which is generally non-stoichiometric), thereby enhancing photoelectrochemical performance. Post-hydrothermal annealing is crucial for achieving high crystallinity, which further reduces the defect density. However, excessive doping can block active sites, underscoring the importance of optimizing Fe precursor concentrations.^60^ Our studies have shown that varying Fe precursors (of the same molarity) and altering annealing atmospheres (vacuum vs air) at the same temperatures can lead to differences in photoelectrochemical behavior due to variation in doping levels and cocatalyst formation. This highlights the necessity of carefully designing heterojunctions to maximize the benefits while minimizing potential drawbacks. The inset shows the CB and VB as estimated from their flat band potentials (which approximately indicate CB for an n-type semiconductor) and by using band gap values derived from the Tauc plot. The CB and VB levels after surface modification are raised compared to that of WO_3_ which is possibly a combined result of Fe doped WO_3_ and Fe_3_O_4_–FeWO_4_ phase formation along with WO_3_. A similar trend is observed in the valence band spectra obtained during XPS measurement, as shown in Figure S7, indicating that the VB maximum of WO_3_/NH_3_/Fe is positioned ∼ 0.7 eV higher than that of pristine WO_3_. But both of these level estimations include the effect of W substrate and also the primary WO_3_ in case of the overlayer. Therefore, the mechanism of electron transfer at Fe_3_O_4_–FeWO_4_/Fe doped WO_3_ heterojunction under illumination may be better visualized with band diagram constructed using values for pure WO_3_, FeWO_4_ and Fe_3_O_4_ taken from literature^35,64^ and presented in Figure 7b. The Fe doping is very low, and therefore, although the VB level should be raised slightly (<0.2 eV, as UV-VisDRS suggests) compared to pure oxide, the values for pure WO_3_ have been used here for mechanistic insight. Electron–hole pairs are created in these semiconductors upon light irradiation of wavelengths smaller than their absorption edge. Low band gap Fe_3_O_4_ may act as a light sensitizer by injecting electrons to the CB of WO_3_. However, it can also create a recombination pathway (dotted line) for electrons to recombine with holes in the VB of Fe_3_O_4_ (which is not at a favorable energy level for water oxidation) and in best case, this could help in Z-scheme charge separation.^65^ The undesired recombination can be mitigated if electrons transfer efficiently from the photoanode to the cathode via the W substrate. To achieve this, employing a direct in situ growth method on the WO_3_ electrode facilitates improved interfacial contact and lattice matching with the tungstate overlayer. The hydrothermal process concurrently introduces Fe dopants into the WO_3_ layer, where unsaturated d orbitals of Fe can provide intermediate energy levels, enhancing charge transfer. Notably, wet-chemical doping directly on WO_3_ electrodes can result in a gradient doping profile due to the element diffusion,^66^ which aids in efficient electron transport through multiple energy steps. The doping in metal oxide like WO_3_ induces the formation of oxygen vacancies, which act as donor states, increasing carrier density and leading to enhanced band bending at the semiconductor/electrolyte interface. This band bending is crucial for efficient charge separation, as it creates an internal electric field that drives photogenerated charge carriers toward the respective electrodes, thereby improving photoelectrochemical performance. Additionally, it is to be noted that the crystalline Fe_3_O_4_ phase likely plays a minimal role in this process due to its relatively low quantity, as indicated by XRD analysis. The VB levels of WO_3_ and FeWO_4_ are favorably below the water oxidation potential just like the operation as a water splitting photoanode demands. The step like heterojunction (type II) forming between WO_3_ and FeWO_4_ helps to prevent recombination by quick charge separation.^16^ The electrons are transferred from higher to lower CB (WO_3_) and the holes are transferred from lower to higher VB (FeWO_4_), from where the electrons can go to external circuit via substrate to cathode for water reduction, and the holes can take part in water oxidation.

**Figure 7:**
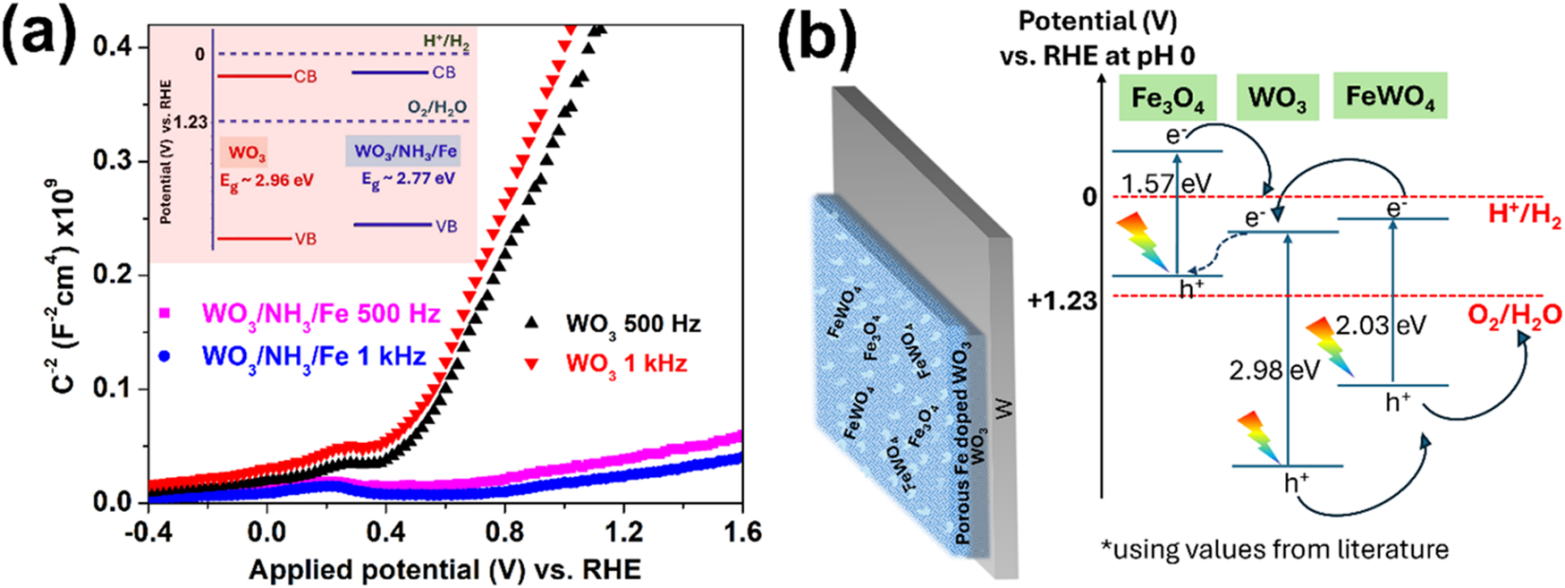
(a) Mott–Schottky plots for WO_3_ and WO_3_/NH_3_/Fe at 500 and 1000 Hz, recorded in the dark and (inset) their approximately estimated conduction and valence band edges. (b) Schematic representation of the composition of WO_3_/NH_3_/Fe photoelectrode and a possible electron–hole separation and transfer mechanism for water oxidation under illumination.

In general, the performance of photoelectrodes is highly dependent on the synthesis procedure. Numerous studies^29,67–69^ have investigated Fe-based overlayers on WO_3_ electrodes synthesized via various methods (Table S3, Supporting Information). Most of these reports demonstrate a photocurrent enhancement comparable to that observed in this work and typically involve multistep procedures based on a transparent substrate, unlike the opaque W substrate used here, which is designed for back illumination. However, there are only a few reports of anodically grown WO_3_ photoanodes with various overlayers/modifications for enhanced photoelectrochemical (PEC) water oxidation.^23,38,40,41,70^ A comparative analysis of these studies is presented in Table S4 (Supporting Information).

## 4 Conclusions

We have successfully developed porous tungsten oxide-based photoelectrodes by using the reproducible technique of anodic tungsten oxidation, followed by a hydrothermal treatment. This resulted in the simultaneous formation of a thin iron tungstate/magnetite based layer on WO_3_ along with doping of WO_3_ with iron. This study is one of the few works devoted to anodized WO_3_ photoelectrodes with overlayers and may inspire future research utilizing such opaque conducting substrates, where light cannot be incident through the WO_3_ side. A slow yet straightforward hydrothermal reaction enables moderately scalable engineering of the crystalline phase on primary electrodes, offering considerable purity and size control at moderate temperatures. However, the vigorous reaction in a liquid medium may damage the primary electrode. Nevertheless, we could find suitable parameters of the one-step hydrothermal engineering of anodic WO_3_ surface with iron based precursor which is critical for applicability as an improved stable photoelectrode under front illumination. Stable photocurrent densities at low bias under simulated solar light were nearly double those of the pristine WO_3_ electrodes. The bang gap change was minimal, and the onset potential remained largely unchanged. Therefore, the observed performance enhancement can be attributed to increased donor density, the presence of oxygen vacancies, and improved charge transfer efficiency due to reduced recombination at the heterojunction. The modifications to WO_3_ also resulted in considerably higher incident photon-to-current conversion efficiencies under visible light, although the improvements were less pronounced in the ultraviolet region. Nonetheless, this method offers a scalable and cost-effective approach to addressing recombination issues in anodized WO_3_ photoelectrodes.

## Supplementary Material



## Data Availability

The data presented in this study are available at RODBUK Cracow Open Research Data Repository at https://doi.org/10.57903/UJ/X9Y5BR

## Notes

Schematic of the optimized route for electrode fabrication, all of the equations used to plot the graphs and explain the results; comprehensive table with details of other samples presented for optimization; FESEM top view image of all samples for optimization except the optimized WO_3_/NH_3_/Fe and WO_3_; LSV curves for these samples, amperometric curves (at 1.2 V vs RHE and 1.0 V vs RHE) for selected samples; charge separation and injection efficiency plots for WO_3_/NH_3_/Fe and WO_3_; XPS valence band spectra of WO_3_/NH_3_/Fe and WO3; performance comparison tables with relevant studies on modified anodic WO_3_ and Fe-modified WO_3_ photoanodes (PDF)
