# Supplementary material for: Hydrothermal Surface Engineering of Anodic WO3 Photoelectrode by Simultaneous Iron Doping and Fe3O4/FeWO4 Formation
Source: ACS Appl Mater Interfaces. 2025 May 8;17(20):30284–96. doi: 10.1021/acsami.5c03437 (PMC12100596; doi:10.1021/acsami.5c03437)
Supplement: Supplementary file 1 [file am5c03437_si_001.pdf]

Supporting Information

Hydrothermal Surface Engineering of Anodic WO_3 Photoelectrode by Simultaneous Iron Doping and Fe_3O_4/$FeWO_4$ Formation

Piyali Chatterjee[a]*, Daniel Piecha[a,b], Sebastian Kotarba[a], Karolina Syrek[a], Marcin Pisarek[c], Grzegorz D. Sulka[a]*

[a]*Department of Physical Chemistry and Electrochemistry, Faculty of Chemistry, Jagiellonian University, Gronostajowa 2, 30-387, Krakow, Poland.*

[b]*Doctoral School of Exact and Natural Sciences, Jagiellonian University, Lojasiewicza 11, 30-348 Krakow, Poland*

[c]*Laboratory of Surface Analysis, Institute of Physical Chemistry, Polish Academy of Sciences, Kasprzaka 44/52, Warsaw 01-224, Poland*

*Corresponding authors. Email: sulka@chemia.uj.edu.pl (G.D.S), piyali93physics@gmail.com (P.C), piyali.chatterjee@uj.edu.pl (P.C).

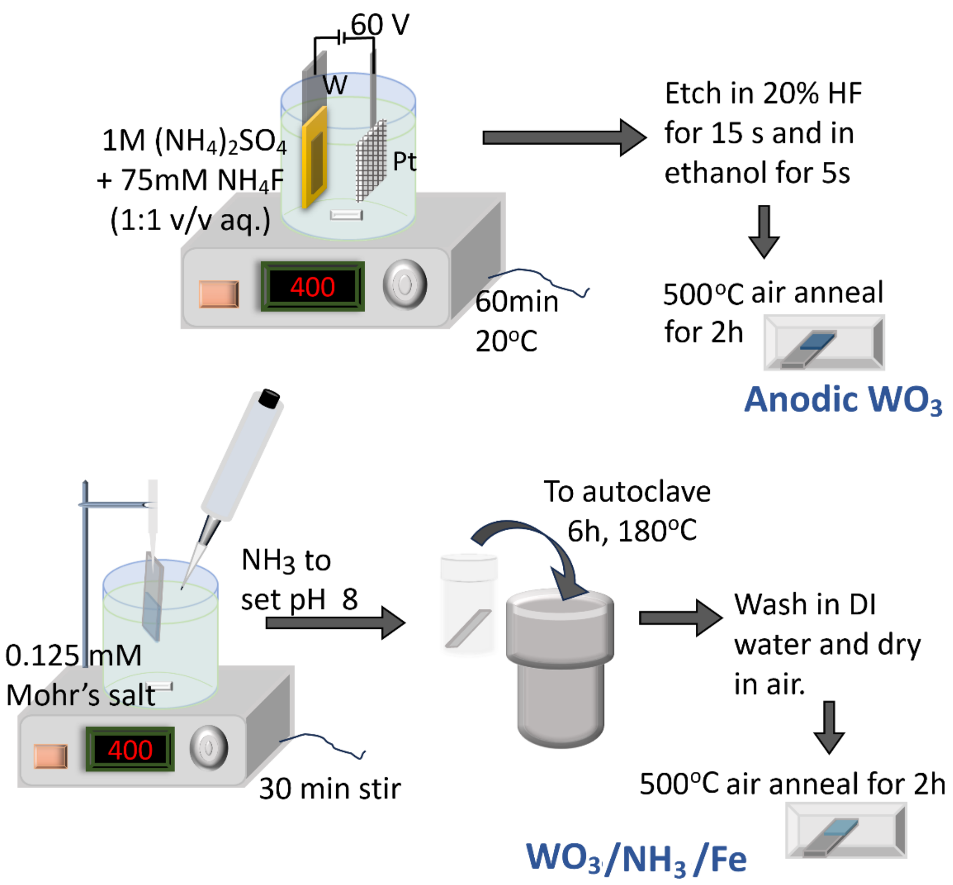

Figure S1: A schematic representation of the synthesis procedure used for the formation of $WO_3/NH_3/Fe$.

A possible reaction pathway for $FeWO_4$ formation, assuming the thermodynamical conditions are favorable, is presented below.

$$2NH_4OH + WO_3 \rightarrow (NH_4)_2WO_4 + H_2O\ (at\ \ the\ surface) \quad \text{S1}$$

$$(NH_4)_2WO_4 + [(NH_4)_2SO_4 \cdot (FeSO_4 \cdot 6H_2O)] \rightarrow FeWO_4 + 2(NH_4)_2SO_4 + 6H_2O \quad \text{S2}$$

Table S1 : Comprehensive list of samples and their hydrothermal treatment conditions.

Sample label	Hydrothermal treatment					Annealing conditions
	Fe precursor	Molarity	Agent for alkaline media	Treatment time (h)	Tempera-ture (°C)	
WO_3						500 °C, 2 h, air
WO_3/NH_3			25% ammonia	6	180	500 °C, 2 h, air
$WO_3/NH_3/Fe$ ($FeSO_4$)	$FeSO_4$	0.125 mM	25% ammonia	6	180	500 °C, 2 h, air
$WO_3/NH_3/Fe$ ($FeCl_3$)	$FeCl_3$	0.125 mM	25% ammonia	6	180	500 °C, 2 h, air
$WO_3/NH_3/Fe$ ($FeCl_2$)	$FeCl_2$	0.125 mM	25% ammonia	6	180	500 °C, 2 h, air
$WO_3/NH_3/Fe$ (0.25 mM)	Mohr's salt	0.25 mM	25% ammonia	6	180	500 °C, 2 h, air
$WO_3/NH_3/Fe$ (0.062 mM)	Mohr's salt	0.062 mM	25% ammonia	6	180	500 °C, 2 h, air
$WO_3/NH_3/Fe$	Mohr's salt	0.125 mM	25% ammonia	6	180	500 °C, 2 h, air
$WO_3/NaOH/Fe$	Mohr's salt	0.125 mM	1 M NaOH	6	180	500 °C, 2 h, air
$WO_3/NH_3/Fe$ (vac)	Mohr's salt	0.125 mM	25% ammonia	6	180	500 °C, 2 h, vacuum

Potentials versus SCE (E_{SCE}) were converted to the corresponding values (E_{RHE}) for the reversible hydrogen electrode (RHE) using the Nernst equation S3 given below:

$$E_{RHE} = E_{SCE} + 0.059pH + 0.219 \quad S3$$

The Debye Scherrer equation S4 used to calculate average crystallite size is given below,

$$D = \frac{0.9\lambda}{B\cos\theta} \quad S4$$

where, D is the crystallite size, 0.9 is a shape factor constant relevant to nanoparticles, B is the full width at half maximum, λ is the wavelength of X-ray radiation, and θ is the Bragg's diffraction angle.

The ABPE% was calculated using equation S5 below, where V_{app} is the applied potential (V) vs. RHE, J is the photocurrent density (mA cm^{-2}), and P_{in} is the power density (mW cm^{-2}) of the incident light.

$$ABPE\% = J(1.23\ V - V_{app}) / P_{in} \quad S5$$

The charge separation efficiency (η_{sep}) was calculated as follows,

$$\eta_{sep} = J_{sulfite}/J_{abs} \quad S6$$

Here, $J_{sulfite}$ refers to the photocurrent density measured in the presence of 0.1 M sodium sulfite added to the electrolyte. J_{abs} represents the maximum possible photocurrent that could be obtained if all absorbed photons were converted into current. This value depends on the UV-visible absorbance spectra of the materials and the spectral intensity of the incident light.

The Tauc equation used to estimate the indirect band gaps is given below in the following equation S7:

$$(Fh\nu)^{\frac{1}{2}} = A(h\nu - E_g) \quad S7$$

where, h, ν, E_g, A, and F are the Planck's constant, the frequency of incident photon, the optical band gap, a material-specific proportionality constant, and the Kubelka-Munk function (absorbance analog) respectively.

The IPCE% is calculated using equation S8:

$$IPCE\% = \frac{1240\, I(\lambda)}{P(\lambda)\, \lambda} \cdot 100\% \quad S8$$

Here, I and P represent the photocurrent density (μA cm^{-2}) and incident light intensity (μW cm^{-2}), respectively, while λ is the wavelength of the incident photon (nm).

The equation S9 as used for Mott-Schottky plot is provided below,

$$\frac{1}{C^2} = \frac{2}{eA^2 \in \in_0 N_D}\left(V - V_{fb} - \frac{TK_B}{e}\right) \quad \text{S9}$$

Here, C and A represent the interfacial capacitance and area, respectively, while N_D is the donor density, V is the applied potential, and V_{fb} is the flat band potential. $\in, \in_0$, K_B, T, and e denote the dielectric constant, permittivity of free space, Boltzmann constant, absolute temperature, electron charge, respectively, as per standard definitions.

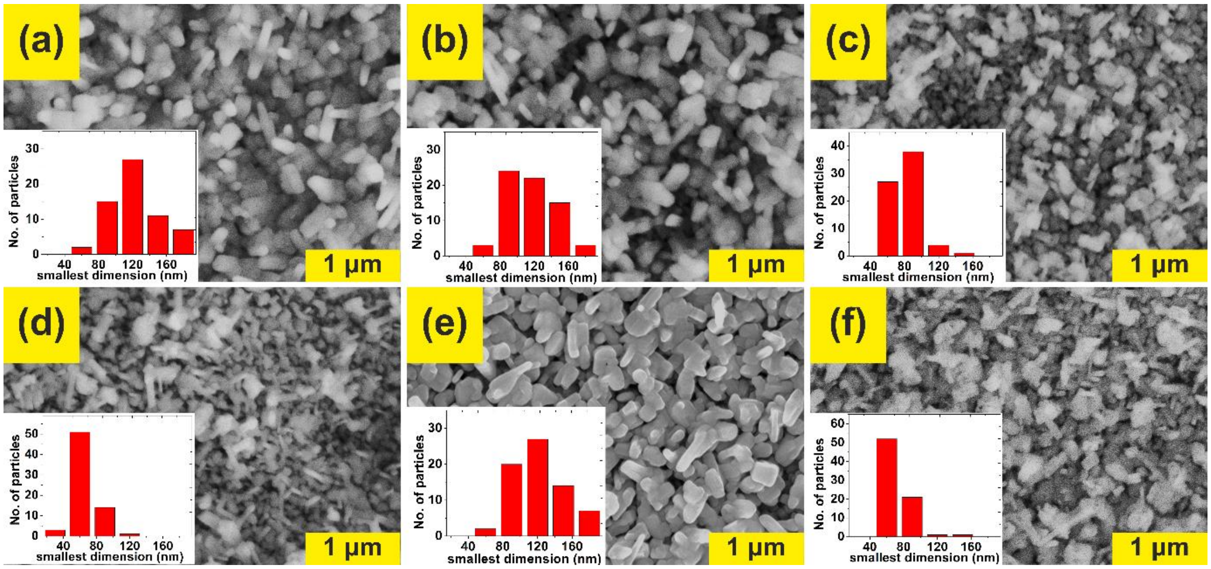

Figure S2: FESEM (top view) images and (inset) their corresponding particle size histograms for (a) WO_3/NH_3, (b) WO_3/NH_3/Fe ($FeSO_4$), (c) WO_3/NH_3/Fe (vac), (d) WO_3/NH_3/Fe (0.25 mM Mohr's salt), (e) WO_3/NH_3/Fe ($FeCl_3$), and (f) WO_3/NaOH/Fe.

FESEM of various samples presented in Figure S2 revealed a mixed morphology, featuring rod-like or thick plate-like structures with significant variations in dimension. Treatment with ammonia alone (Figure 2S(a)) and the use of $FeCl_3$ or $FeSO_4$ as iron precursors (Figure S2(b) and S2(e))

result in larger average particle sizes compared to those shown in Figure S2(c), S2(d), and S2(f), which are synthesized using the Mohr's salt. Notably, treatment with NH_3 alone caused degradation of the WO_3 electrode surface, leading to the re-precipitation of very large particles. As a result, this material was excluded from further consideration and discussion in this study. The histograms of the samples (insets in Figure 2S) indicate that the use of the Mohr's salt at very low molarity (e.g., $WO_3/NH_3/Fe$ (vac) and $WO_3/NaOH/Fe$) yields smaller mean particle sizes with a narrower size distribution, measured by the smallest dimensions of the irregular structures observed. The smaller overlayer particle size probably contributes to higher surface area and potentially improve the accessibility of WO_3 pores, making these samples more favorable for co-catalytic applications. An alternative Fe^{2+} precursor, $FeCl_2$, was also tested for the hydrothermal modification of anodic WO_3; however, the resulting photoanodes exhibited very low photocurrents under white light illumination. Its corresponding FESEM image (Figure S3) shows the formation of excessively large agglomerated particles on the surface.

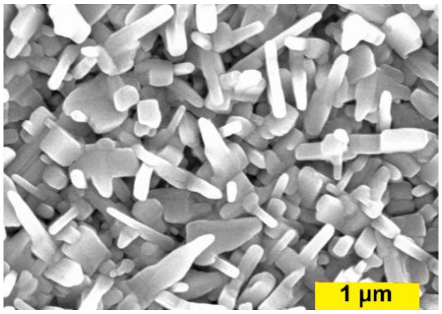

Figure S3: FESEM top view image of $WO_3/NH_3/Fe$ ($FeCl_2$).

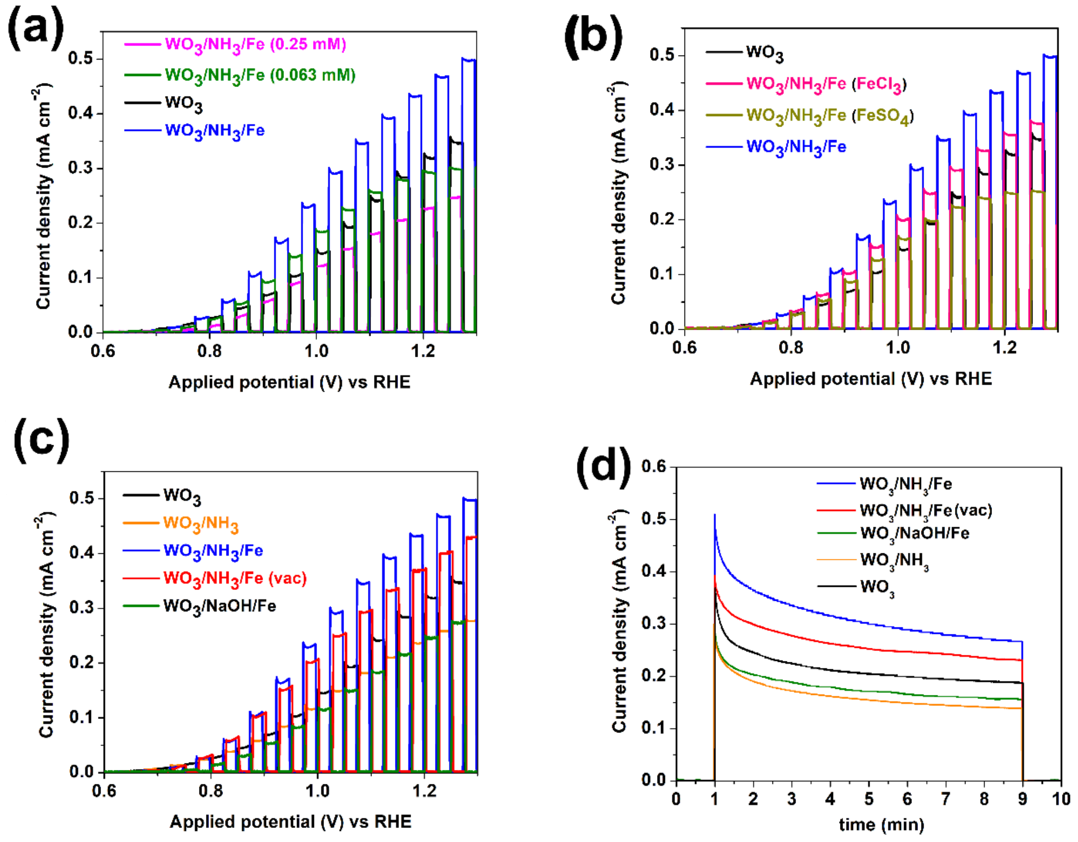

Figure S4: Chopped LSVs under 1.5 AM G illumination for WO_3 and the optimised $WO_3/NH_3/Fe$ sample (synthesized with 0.125 mM Mohr's salt and ammonia, followed by air annealing) is compared against (a) electrodes synthesized with 0.25 mM and 0.063 mM Mohr's salt; (b) electrodes synthesized using $FeCl_3$ and $FeSO_4$ as precursors (instead of the Mohr's salt); (c) electrodes synthesized without an Fe precursor, with NaOH (instead of NH_3); annealed in vaccuum (instead of air). (d) Current vs. time curves at 1.2 V vs. RHE for $WO_3/NH_3/Fe$, $WO_3/NH_3/Fe$ (vac), $WO_3/NaOH/Fe$, and WO_3/NH_3 compared to WO_3.

Table S2: Comparison of resistive parameters obtained from equivalent circuit fitting of Nyquist plots recorded at 1 V vs. RHE.

	**WO_3** 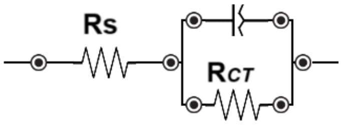		**$WO_3/NH_3/Fe$** 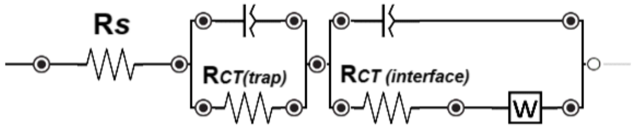			
	R_s Ohm.cm^2	R_{CT} Ohm.cm^2	R_s Ohm.cm^2	$R_{CT\ (trap)}$ Ohm.cm^2	$R_{CT(interface)}$ Ohm.cm^2	Warburg element σ
**light**	14.7	1899.6	18.9	88.7	546.5	75

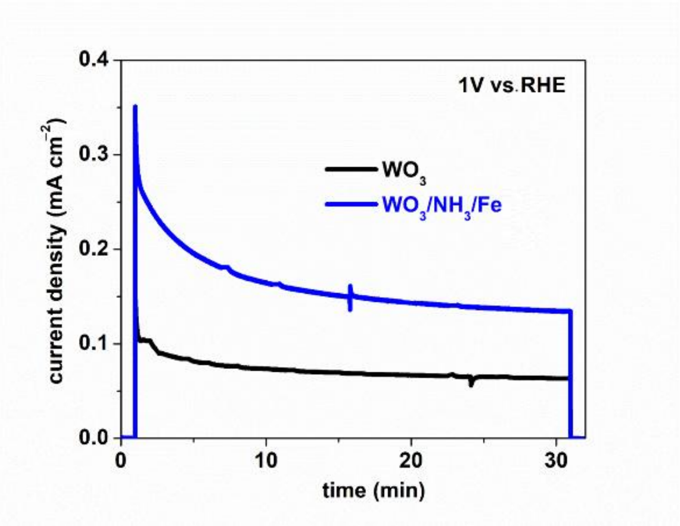

Figure S5: Current vs time curves at 1 V vs. RHE for a different batch of fresh $WO_3/NH_3/Fe$ and WO_3 samples for 30 min of illumination at 1 V vs. RHE fixed bias.

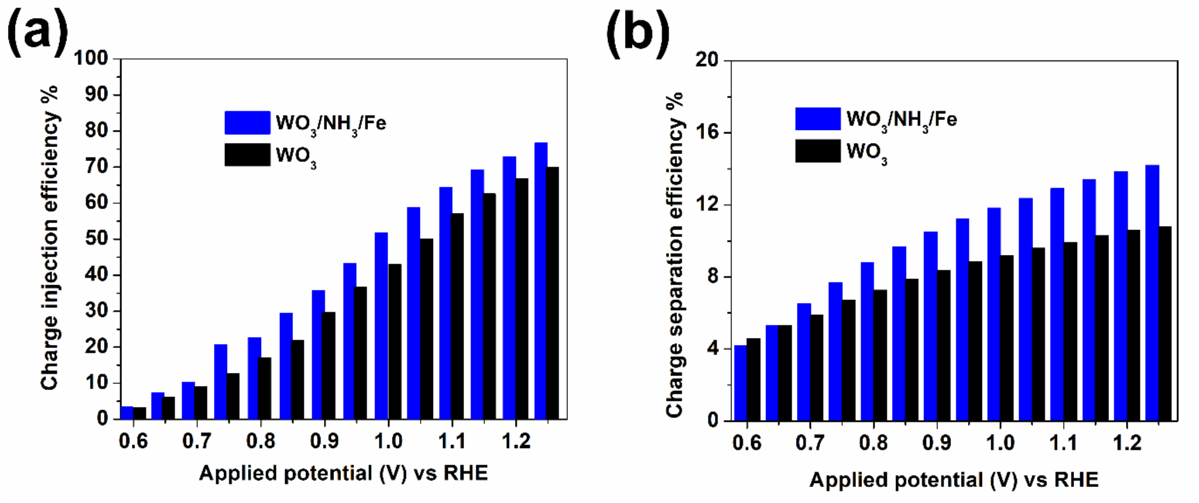

Figure S6: (a) Charge injection efficiency and (b) charge separation efficiency for $WO_3/NH_3/Fe$ and WO_3.

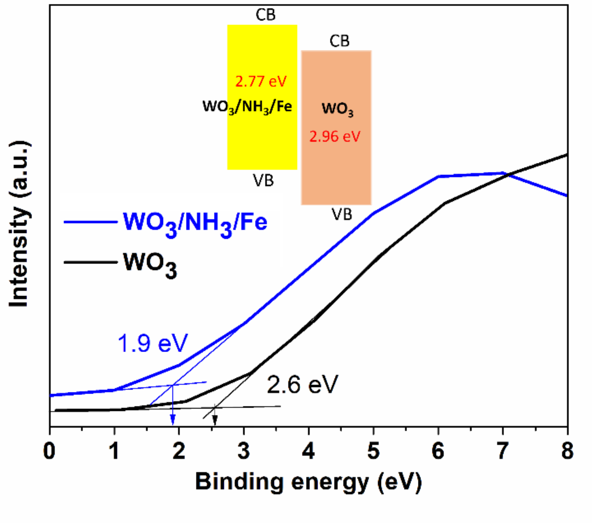

Figure S7: Valence band spectra from XPS for the $WO_3/NH_3/Fe$ and WO_3 electrodes and corresponding band diagram (inset).

Table S3: Listing and performance comparison of WO_3-based photoanodes fabricated using methods other than anodic oxidation, with Fe-based overlayers.

**Electrode composition**	**WO_3 synthesis method**	**Fe based overlayer synthesis method**	**Electrolyte**	**Change in photocurrent density compared to WO_3 (under solar illumination)**	**Ref. No.**
FTO/WO_3/ Fe_2O_3	Hydrothermally grown WO_3	Spin coating Fe_2O_3 precursor	(Not found)	~2 times higher at 1.2 V and 1.5 times higher at 1 V vs. RHE	1
FTO/WO_3/ Fe_2O_3	Drop-casted WO_3 precursor	Electrodeposited Fe_2O_3	0.2 M Na_2SO_4	~8 times higher at 1.2 V vs. RHE	2
FTO/WO_3/ Fe_2O_3	Hydrothermally grown WO_3	Immersed in heated Fe precursor	Potassium phosphate buffer (pH=7)	~2 times higher at 1.2 V vs. RHE	3
FTO/WO_3/ Fe2O_3	Spin coated WO_3 seed layer + hydrothermally grown WO_3	Hydrothermally grown Fe_2O_3	0.1 M Na_2SO_4	1.6 times higher at 1.2 V vs. RHE	4
FTO/WO_3/ Fe_2O_3	WO_3 (hydrothermally synthesized) coated by doctor-blade method	Deposition-annealing Fe(III) precursor	0.2 M Na_2SO_4	~1.8 times higher at 1.2 V vs. RHE	5
FTO/WO_3/ FeOOH	Spray-pyrolyzed WO_3	Electrodeposited FeOOH	0.5 M K_2SO_4	~4 times higher at 1 V vs. RHE (front illumination)	6
FTO/WO_3 / FeOOH	Spin-coated WO_3 precursor + hydrothermally grown WO_3	Photo-deposited thin FeOOH layer	0.1 M potassium phosphate buffer (pH = 7)	~2.2 times higher at 1.2 V vs. RHE	7
FTO/WO_3/ Fe_2O_3	Spin-coated WO_3 precursor + hydrothermally grown WO_3	Thermally decomposed electrodeposited Prussian blue	0.1 M Na_2SO_4	7 times higher at 1.2 V vs. RHE	8
FTO/WO_3/ Fe_2O_3	Hydrothermally grown WO_3	Hydrothermally grown Fe_2O_3	0.1 M potassium phosphate buffer (pH = 7)	1.6 times higher at 1.2 V vs. RHE	9

FTO/WO_3/ Fe_2O_3/ FeOOH	Hydrothermally grown WO_3	Hydrothermally grown Fe_2O_3 + chemical bath deposition of FeOOH	0.2 M Na_2SO_4	3.73 times higher, (but 2 times higher without FeOOH) at 1.2 V vs. RHE	10
FTO/WO_3/ phases with Fe(II/III) oxidation states	Drop-casted WO_3 (hydrothermally synthesized)	Fe(II) precursor deposition-annealing	0.1 M Na_2SO_4	~3 times higher at 1.2 V vs. RHE	11
FTO/WO_3/Fe-doped WO_3/ Fe_2WO_6 / FeOOH	Spin-coated WO_3 precursor + hydrothermally grown Fe doped WO_3	Spray-pyrolysis Fe_2WO_6 + FeOOH photo-electro-deposition	0.5 M phosphate buffer (pH=7)	~2 times higher at 1 V and 1.2 V vs. RHE	12
W/Fe-doped WO_3/$FeWO_4$/ Fe_3O_4	Anodic oxidation of WO_3	One step hydrothermal treatment by Fe(II) precursor	0.1 M Na_2SO_4	~2 times higher at ~1 V vs. RHE and ~1.3 times higher at 1.2 V vs. RHE (front illumination)	This work

Table S4: A comparison of anodic WO_3 photoelectrodes with a overlayer or cation doping for PEC water oxidation.

**Electrode composition on W substrate**	**WO_3 modification method**	**Electro-lyte**	**Summary of change in photocurrent density compared to anodic WO_3**	**Perform-ance under visible light**	**Ref. No.**
WO_3/MoS_x	(One-step fabrication) plasma electrolytic W oxidation	0.1 M Na_2SO_4 (pH= 2)	2 times higher at ~1.23V vs. RHE under light of 365 nm wavelength		13
Co-doped WO_3	(one-step fabrication) Anodic W oxidation in electrolyte with Co-precursor	0.1 M KNO_3	~3.5 times higher at ~1.2 V vs. RHE under solar simulated light, 10 times lower under UV	7 times higher IPCE% under 500 nm light	14

WO_3/ $CuWO_4$	Wet impregnation	0.1 M KNO_3	Worse under UV	IPCE% higher under light of 450-490 nm wavelength	15
WO_3/Bi_2S_3	SILAR method	0.1 M Na_2S/ Na_2SO_3 (pH=12.49)	19 times higher at 0.95V vs. RHE under solar simulated light (Note: Electrode undergoes complete corrosion in some minutes.)		16
WO_3/ $BiVO_4$	Spin coating	0.5 M Na_2SO_4	2.4 times higher at 1.2 V and ~1.3 times at 1 V vs. RHE) under solar simulated light	IPCE% extended up to light of 480 nm wavelength (~50 nm higher)	17
WO_3/Fe_2O_3	Electrodeposition	0.1 M KNO_3	IPCE% worse under light of up to 450 nm wavelength	IPCE% significantly high, extended up to light of 550 nm wavelength (~70 nm higher)	18
WO_3/ $FeWO_4$/ Fe_3O_4/Fe-doped WO_3	Hydrothermal treatment	0.1 M Na_2SO_4	2 times higher at ~1 V and ~1.3 times higher at 1.2 V vs. RHE under solar simulated light	IPCE% ~5 times higher under light of 450 nm wavelength	This work

References:

(1) Fan, X.; Wang, T.; Xue, H.; Gao, B.; Zhang, S.; Gong, H.; Guo, H.: Song, Li.; Xia, W.; He, J. Synthesis of tungsten trioxide/hematite core-shell nanoarrays for efficient photoelectrochemical water splitting. *ChemElectroChem* **2018**, *6* (2), 543–551. DOI: 10.1002/celc.201801181.

(2) Mao, A.; Kim, J.K.; Shin, K.; Wang, D.H.; Yoo, P. J.; Han, G.Y.; Park, J.H. Hematite modified tungsten trioxide nanoparticle photoanode for solar water oxidation. *J. Power Sources* **2012**, *210*, 32–37. DOI: 10.1016/j.jpowsour.2012.02.112.

(3) Davi, M.; Ogutu, G.; Schrader, F.; Rokicinska, A.; Kustrowski, P.; Slabon, A. Enhancing photoelectrochemical water oxidation efficiency of WO_3/α-Fe_2O_3 heterojunction photoanodes by surface functionalization with CoPd nanocrystals. *Eur. J. Inorg. Chem.* **2017**, *2017* (37), 4267–4274. DOI: 10.1002/ejic.201700952.

(4) Zheng, G.; Jiang, S.; Cai, M.; Zhang, F.; Yu, H. WO_3/FeOOH Heterojunction for improved charge carrier separation and efficient photoelectrochemical water splitting. *J. Alloy. Compd.* **2024**, *981*, 173637–173637. DOI: 10.1016/j.jallcom.2024.173637.

(5) Han, S.; Li, J.; Chen, X.; Huang, Y.; Liu, C.; Yang, Y.; Li, W. enhancing photoelectrochemical activity of nanocrystalline WO_3 electrodes by surface tuning with Fe(III). *Int. J. Hydrogen Energ.* **2012**, *37* (22), 16810–16816. DOI: 10.1016/j.ijhydene.2012.08.145.

(6) Kwong, W. L.; Lee, C. C.; Messinger, J. Transparent nanoparticulate FeOOH improves the performance of a WO_3 photoanode in a tandem water-splitting device. *J. Phys. Chem. C* **2016**, *120* (20), 10941–10950. DOI: 10.1021/acs.jpcc.6b02432.

(7) Huang, J.; Ding, Y.; Luo, X.; Feng, Y. solvation effect promoted formation of p–n junction between WO_3 and FeOOH: a high performance photoanode for water oxidation. *J. Catal.* **2015**, *333*, 200–206. DOI: 10.1016/j.jcat.2015.11.003.

(8) Mao, G., Wu, H., Qiu, T., Bao, D., Lai, L., Tu, W., & Liu, Q. WO_3@Fe2O_3 core-shell heterojunction photoanodes for efficient photoelectrochemical water splitting. *Chinese J. Struc. Chem.* **2022,** *41* (8), 2208025–2208030. DOI: 10.14102/j.cnki.0254-5861.2022-0086

(9) Jin, T.; Diao, P.; Wu, Q.; Xu, D.; Hu, D.; Xie, Y.; Zhang, M. WO_3 nanoneedles/α-Fe_2O_3/cobalt phosphate composite photoanode for efficient photoelectrochemical water splitting. *Appl. Catal. B- Environ.* **2014**, *148-149*, 304–310. DOI: 10.1016/j.apcatb.2013.10.052.

(10) Zhang, J.; Zhu, G.; Liu, W.; Xi, Y.; Golosov, D. A.; Zavadski, S. M.; Melnikov, S. N. 3D core-shell WO_3@α-Fe_2O_3 photoanode modified by ultrathin FeOOH layer for enhanced

photoelectrochemical performances. *J. Alloy. Compd.* **2020**, *834*, 154992. DOI: 10.1016/j.jallcom.2020.154992.

(11) Chatterjee, P.; Chakraborty, A. K. Enhanced photoelectrochemical water oxidation by Fe(II) modified nanostructured WO_3 photoanode. *Opt. Mater.* **2023**, *144*, 114361. DOI: 10.1016/j.optmat.2023.114361.

(12) Lin, H.; Long, X.; An, Y.; Yang, S. *In situ* growth of Fe_2WO_6 on WO_3 nanosheets to fabricate heterojunction arrays for boosting solar water splitting. *J Chem. Phys.* **2020**, *152* (21), 214704. DOI: 10.1063/5.0008227.

(13) Levinas, R.; Tsyntsaru, N.; Cesiulis, H.; Viter, R.; Grundsteins, K.; Tamašauskaitė-Tamašiūnaitė, L.; Norkus, E. Electrochemical synthesis of a WO_3/MoS_x heterostructured bifunctional catalyst for efficient overall water splitting. *Coatings* **2023**, *13* (4), 673. DOI: 10.3390/coatings13040673.

(14) Syrek, K., Kotarba, S., Zych, M., Pisarek, M., Uchacz, T., Sobańska, K., Pięta Ł., Sulka, G. D. Surface engineering of anodic WO_3 layers by in situ doping for light-assisted water splitting. *ACS Appl. Mater. Interfaces* **2024**, *16* (28), 36752-36762. DOI: 10.1021/acsami.4c02927

(15) Zych, M.; Syrek, K.; Wiercigroch, E.; Malek, K.; Kozieł, M.; Sulka, G. D. Visible-light sensitization of anodic tungsten oxide layers with $CuWO_4$. *Electrochim. Acta* **2021**, *368*, 137591. DOI: j.electacta.2020.137591.

(16) Abouelela, M. M.; Kawamura, G.; Tan, W. K.; Matsuda, A. Anodic nanoporous WO_3 modified with Bi_2S_3 quantum dots as a photoanode for photoelectrochemical water splitting. *J. Colloid Interf. Sci.* **2023**, *629*, 958–970. DOI: 10.1016/j.jcis.2022.09.041.

(17) Park, E.; Yoo, J.; Lee, K. Enhanced photoelectrochemical hydrogen production *via* linked $BiVO_4$ nanoparticles on anodic WO_3 nanocoral structures. *Sustain. Energ. Fuels* **2024**, *8* (7), 1448–1456. DOI: 10.1039/d3se01545a.

(18) Zych, M.; Syrek, K.; Pisarek, M.; Sulka, G. D. Anodic WO_3 layers sensitized with hematite operating under the visible light spectrum. *J. Power Sources* **2022**, *541*, 231656. DOI: 10.1016/j.jpowsour.2022.231656.
